# A Case of a Patient With *MYH2*-Associated Myopathy Presenting With a Chief Complaint of Hand Tremor

**DOI:** 10.5334/tohm.932

**Published:** 2024-10-01

**Authors:** Xinxin Liao, Qiuxiang Li, Huan Yang, Qiying Sun

**Affiliations:** 1 Department of Geriatric Neurology, Xiangya Hospital, Central South University, Changsha, Hunan 410008, China; 2 National Clinical Research Center for Geriatric Disorders, Changsha, Hunan 410078, China; 3 Department of Neurology, Xiangya Hospital, Central South University, Changsha, Hunan 410008, China

**Keywords:** myogenic tremor, myopathy, ophthalmoplegia, myosin, *MYH2*

## Abstract

**Background:**

Postural tremor is an uncommon and often overlooked phenotype in skeletal myopathy, which may lead to diagnostic delays.

**Case report:**

A 21-year-old man presented with adolescent onset postural hand tremor as the initial symptom, followed by mild limb muscle weakness. Neurological examination showed restricted ocular motility without diplopia and myopathic facial appearance. A muscle biopsy showed a decrease in type 2A fibers. Whole-exome sequencing identified two novel compound heterozygous variants in *MYH2* gene (NM_017534.6): c.505+2T>C and c.3565 del C. The diagnosis was further validated via bioinformatics analysis and confirmed through familial co-segregation by Sanger sequencing.

**Discussion:**

This report expands the mutational and phenotypic spectrum of *MYH2*-associated myopathy. We suggest that in the differential diagnosis of tremor, besides common neurogenic causes, myogenic etiology should also be considered.

**Highlights:**

Hand tremor in this case expands the phenotype of MYH2-associated myopathy, enhancing our understanding of tremor origins. It underscores the importance of nuanced clinical assessment and genetic screening in complex tremor disorders.

## Introduction

Tremor is defined as an involuntary, rhythmic, and oscillatory activity that can involve various parts of the body. The generation of rhythmic tremor activity necessitates the presence of “oscillators”, which are functional systems composed of interconnected body parts, including muscles, peripheral nerves, central nuclei, and neuronal networks. The intricate interactions within these oscillator systems can result in a wide range of tremor etiologies. Most tremor syndromes are associated with the central nervous system pacemakers, while peripheral nervous system pacemakers are less commonly implicated. Additionally, myogenic tremor stands out as a recently recognized and potentially under-characterized novel type of tremor, with the tremor pacemaker uniquely situated at the level of the myofibrils [1].

To date, myogenic tremor has only been reported in association with mutations in sarcomere-associated genes, mainly the thick filaments and the thin filaments. Myosin, a major structural component of the thick filaments, is crucial for bodily movement and cardiac contractility [2]. The conventional two-headed myosin is a hexamer formed by two identical myosin heavy chains (MyHCs) and two pairs of non-identical myosin light chains (MyLCs) [3]. In adult skeletal muscles, MyHC IIa, one of the three major MyHC isoforms, is encoded by *MYH2* gene and expressed in fast-type 2 A and 2B muscle fibers. Mutations in the *MYH2* gene lead to a rare group of hereditary myopathies with both autosomal dominant (AD) and recessive (AR) modes of transmission [4,5,6,7].

Herein, we describe a male patient with *MYH2*-associated myopathy who exhibited high-frequency, postural tremor that was notably pronounced in his fingers and appeared in conjunction with muscle weakness and ophthalmoplegia. The diagnosis was confirmed by the identification of two novel compound heterozygous variants in *MYH2* via whole-exome sequencing and a muscle biopsy that revealed a reduction in type 2 A fibers. The co-occurrence of tremor and muscle weakness strongly indicates a sarcomeric origin, offering a valuable clinical indicator for the differential diagnosis of complex tremor disorders.

## Case report

**Figure 1:**
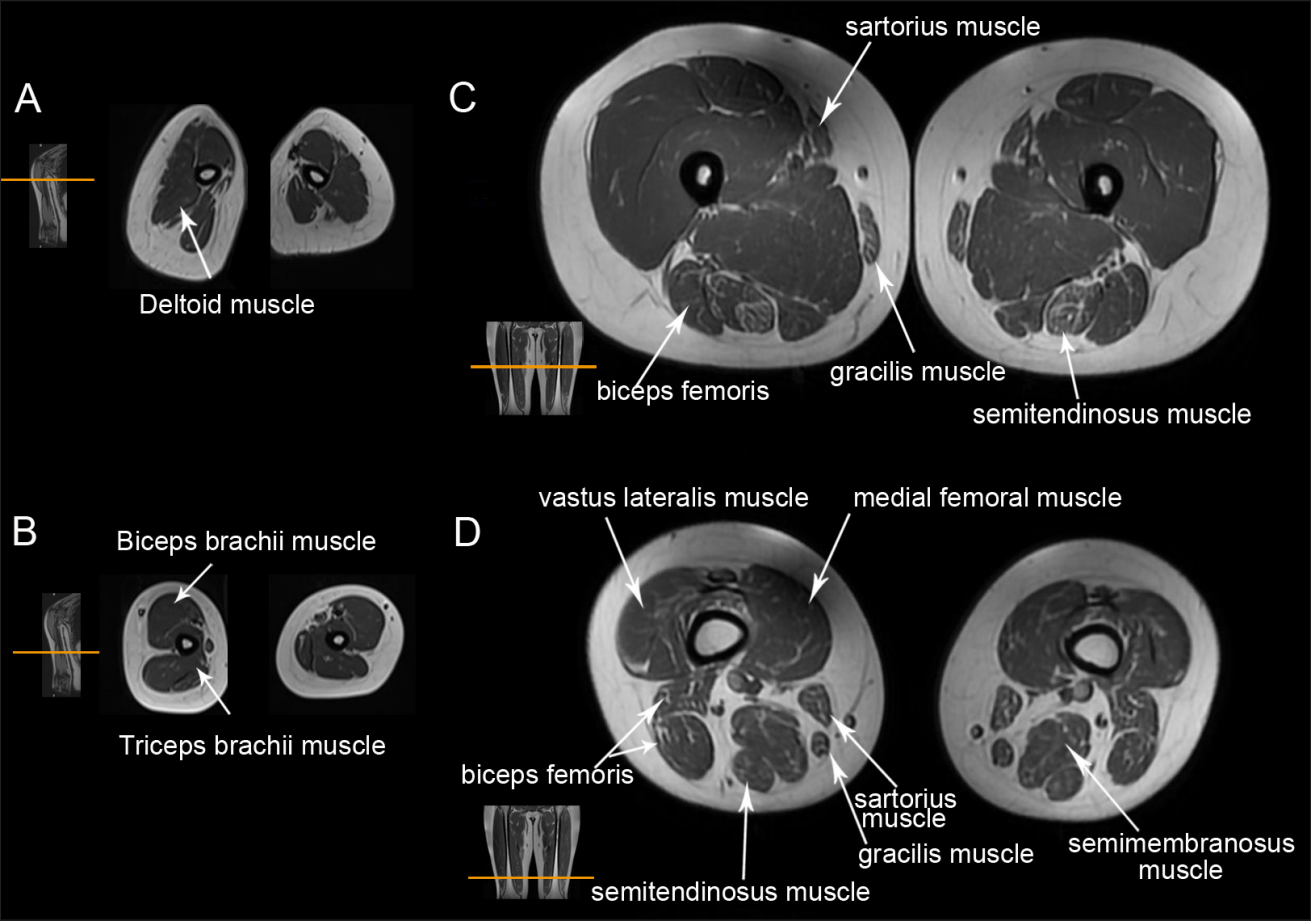
T1-weighted transverse magnetic resonance images of the patient’s arms **(A–B)** and thighs **(C–D)**. Mild fatty infiltration of the deltoid muscle and triceps brachii was observed in the upper arms. Mild-to-moderate fatty infiltration was observed in the biceps femoris, quadriceps femoris, sartorius, semitendinosus, semimembranosus, and gracilis muscles.

The patient was a 21-year-old Chinese man who was the only child of healthy, non-consanguineous parents. He was born at 39 weeks’ gestation via spontaneous vaginal delivery, and exhibited normal motor development with no history of early joint contracture. During his middle school years, he reported involuntary bilateral hand tremor in specific postures, particularly noticeable when pouring water into a narrow-mouthed container. Despite the prior diagnosis of essential tremor and treatment with propranolol, there was no improvement in his symptoms. He later noticed a slight decrease in muscle strength, characterized by diminished explosive power, which became evident during sprint tests. At the age of 21, he had difficulty when lifting heavy objects and visited our clinic for the first time. He had no symptoms of prominent dysphagia. Examination revealed near-complete ophthalmoplegia, slight constant ptosis, and myopathic facial appearance. Muscle strength, graded using the Medical Research Council (MRC) scale, was 4/5 for neck flexor muscles, 4+/5 for shoulder abduction, 4+/5 for elbow flexion, and extension, and 4/5 for hand intrinsics muscles. Mild bilateral facial weakness was observed, characterized by a weak smile and normal eyelid closure strength. Tongue lateralization demonstrated normal agility and range of motion. The strengths of the bilateral hip abductor, knee extensor, and ankle dorsiflexor muscles were all rated as normal. Muscle atrophy was not apparent, except for mild atrophy of the tongue, and mild atrophy of both the thenar and hypothenar muscles. There was a mild, medium to high frequency postural tremor involving the fingers, and the tremor symptoms did not worsen upon the 3-year follow-up examination (Video 1). Spooning was present in both hands when the arms were extended forward, with the left hand showing a more pronounced effect in the wing-beat position. Additionally, there was a clear indication of dystonic flexion in the right fifth finger during the wing-beat position. Extremely mild tremors were observed on the left hand during the spiral drawing test. On the nose-finger-nose test, no intention tremors and dysmetria were observed. No signs of sensory impairment were observed. The patient had a normal gait, with no difficulty walking uphill or climbing stairs. Serum creatine kinase levels ranged between 444 and 812 IU/L (normal range: 50–310 IU/L). His resting-state lactate concentration ranged between 2.87 and 3.18 mmol/L (normal range: 1.42–1.9 mmol/L). Routine blood test, thyroid function tests and serum levels of ceruloplasmin, ferritin and vitamin B12 were within normal limits. Nerve conduction studies and electromyographic findings were unremarkable. The electrocardiogram and echocardiography were normal. Magnetic resonance imaging (MRI) revealed mild fatty infiltration of the deltoid muscle and triceps brachii in the upper arms and the biceps femoris, sartorius, semitendinosus, semimembranosus, and gracilis in the thigh (Figure 1). No abnormalities or atrophy were detected in the brain MRI. Serum creatine kinase levels were normal in both of the patient’s parents.

**Video 1:** Neurological Examination: Initial visit and 3-Year Follow-up. This video demonstrates mild, medium-to-high frequency postural tremors affecting the distal joints of both hands, observed during both the initial visit and the 3-year follow-up. Spooning is present in both hands when the arms are extended forward, with the left hand showing a more pronounced effect in the wing-beat position. Additionally, there is a clear indication of dystonic flexion in the right fifth finger during the wing-beat position. Extremely mild tremors were observed on the left hand during the spiral drawing test. The nose-finger-nose test is negative, suggesting the absence of kinetic tremor.

**Figure 2:**
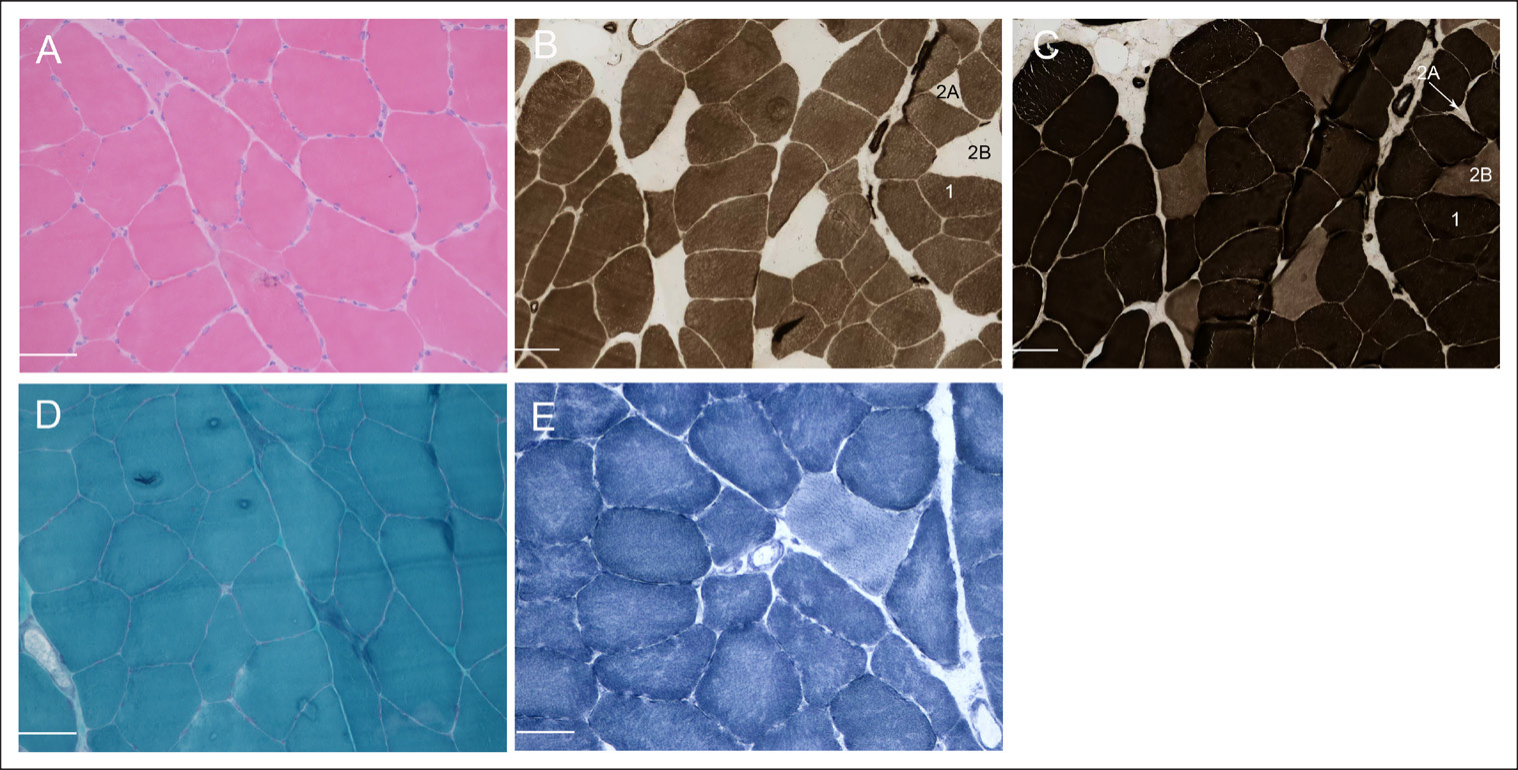
Muscle biopsy from patient’s brachial biceps conducted at 21 years of age. **(A)** Mild fiber size variation is detectable (H&E). **(B–C)** ATPase staining of serial sections at different pH levels: **(B)** ATPase pH 4.2; and **(C)** ATPase pH 4.5. These figures illustrate three different fiber types. Black staining corresponds to type 1 fibers. In both figures B and C, grayish-white staining corresponds to Type 2 A fibers. In figure B, grayish-white staining also indicates Type 2B fibers, whereas in figure C, Type 2B fibers are indicated by dark gray staining. Within the observed field of view, only one small type 2 A fiber was observed (indicated by an arrow). **(D)** Nemaline rod or rod-like structures, filamentous tangles, ragged red fibers, or rimmed vacuoles were not observable via modified Gomori trichrome staining. **(E)** With nicotinamide adenine dinucleotide dehydrogenase-tetrazolium reductase (NADH-TR) staining, the intermyofibrillar network was slightly disturbed. Scale bar, 100 μm.

A biopsy of the *biceps brachii* muscle, conducted at age 21, revealed mild variability in fiber size with scattered atrophic or hypoplastic fibers (Figure 2A). No obvious inflammatory cell infiltration was observed in the endomysium or perimysium. The fiber types were identified as ATPases, indicating marked predominance of type 1 fibers and minimal type 2 fibers (Figure 2B, C). Neither ragged red nor ragged blue muscle fibers were observed via modified Gomori trichrome (Figure 2D) or succinic acid dehydrogenase (SDH) staining, respectively. No Cox-deficient fibers were observed by COX/SDH double-staining. The intermyofibrillar network was slightly disturbed (Figure 2E).

**Figure 3:**
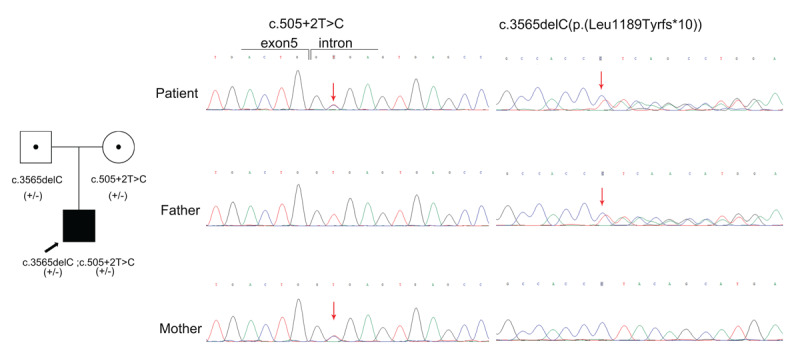
Family pedigree and sequence chromatograms of *MYH2* variants. The localization of variants is indicated by arrows. Squares, males; circles, females. The filled symbols and those with central dots represent the affected individuals and variants carriers, respectively. The genotypes of each family member in the *MYH2* are shown. +, mutant allele; –, wild-type allele. The thick arrow indicates the proband. Identification of two variants in the *MYH2* gene in the proband. First, the heterozygous c.3565 del C variant, which was inherited from the father. The second variant is a heterozygous c.505 + 2T>C splice site variant, which was inherited from the unaffected mother.

Total DNA was isolated from the patient’s *biceps brachii* muscle and from the peripheral blood lymphocytes of his parents. Whole-exome sequencing conducted on the proband identified two heterozygous *MYH2* variants (NM_017534.6): c.505 + 2T>C and c.3565 del C (p. (L1189Yfs*10)). The patient’s entire mitochondrial DNA (mtDNA) genome was enriched by single-amplicon long-range PCR followed by massive parallel sequencing; no pathogenic mtDNA variants, large deletions, or duplications were detected. Sanger sequencing was performed for verification and segregation analysis in the patient and his parents. The mother and father were heterozygous for c.505 + 2T>C and c.3565 del C, respectively (Figure 3). Both variants were absent from all population databases, including gnomAD (version 2.1.1) and Exome Aggregation Consortium (ExAC) database. The maternally inherited variant (c.505 + 2T>C) occurs on a canonical splice donor site of exon 3, which was predicted to be a splice-altering variant with an extraordinary score of 0.9759 on the Splice AI tool (v1.3) (https://github.com/Illumina/SpliceAI) and 36 on CADD v1.7 (CADD: Combined Annotation-Dependent Depletion; https://cadd.gs.washington.edu) [8,9,10]. The thresholds are 0.2 for splice AI and 14.25 for CADD, respectively. The second *MYH2* variant, c.3565 del C, was predicted to result in a frameshift and premature stop codon (p. (L1189Yfs*10)), resulting in truncation of the proximal coiled-coil rod domain—and was therefore predicted to be pathogenic. Both variants were categorized as pathogenic according to the American College of Medical Genetics and Genomics/Association for Molecular Pathology (ACMG/AMP) guidelines [11]: for variant c.505 + 2T>C, PVS1, PM2, PP3, and PP4; for c.3565 del C, PVS1, PM2, and PP4. Given that the patient’s parents showed no symptoms of muscle weakness or tremor, and the genetic testing results were consistent with the principle of co-segregation, the patient was diagnosed with autosomal recessive *MYH2*-associated myopathy.

## Discussion

MyHC IIa myopathies are rare diseases with high levels of clinical and histological heterogeneity. To date, 7 AD and 23 AR mutations in *MYH2* have been reported worldwide. Our patient carried two novel heterozygous variants, and there are several lines of evidence that these two variants are the cause of the congenital myopathy observed in this family: (1) The clinical and pathological phenotypes of the patient matched the typical characteristics of AR-*MYH2* myopathy; (2) Both variants are not reported in any public population database, while non-pathogenic variants in *MYH2* are very rare, considering the strong selective pressure against mutations in this gene as demonstrated by Tajshargi et al. [12]; (3) Fifteen out of 17 *in silico* analyses predicted the c.505 + 2T>C to be pathological, including SpliceAI and CADD; (4) The c.3565 delC variant shifts the reading frame and leads to a premature stop codon p.(L1189Yfs*10)—and a similar recessive single nucleotide deletion variant (c.4352 Adel;p. K1451Sfs*40), which is distal to the current *MYH2* variant, has been previously reported to cause a severe phenotype [13]; (5) Whole-exome sequencing and optimized human mitochondrial genome sequencing did not reveal any other pathogenic variants that could explain the patient’s clinical phenotype.

In accordance with the previous reports, nonsense-mediated decay (NMD) leading to reduced transcript expression is one of the pathogenic mechanisms mediated by the nonsense mutations in *MYH2* [4,6,13]. The two variants identified in our patient are predicted to potentially produce premature termination codons (PTCs), suggesting that transcripts from both mutated alleles might be degraded by NMD. To verify this hypothesis, the cDNA of *MYH2* should be amplified and sequenced to confirm the presence of aberrant transcripts. Additionally, the proportion of each MyHC isoform in skeletal muscle should be quantified. The truncated and potentially non-functional or partially functional protein should also be detected using the MyHC IIa antibody by western blot.

It is worth noting that the chief complaint of our patient is hand tremor related to both posture and action. The tremor is localized to the distal joints (fingers) rather than the more proximal (wrist and elbow) tremor which is typically seen in essential tremor. In some cases, patients may not notice muscle weakness or ocular dysmotility without diplopia, since the clinical presentation is typically mild or static for prolonged periods—particularly among patients with a recessive pattern—resulting in delayed diagnosis. Although hand tremor is not a common clinical feature of *MYH2*-associated myopathy, it has been reported in patients with four distinct *MYH2* gene mutations, encompassing both recessive and dominant types [5,14,15,16]. Furthermore, tremors have frequently been associated with mutations in genes encoding sarcomeric proteins, such as those affecting MyHC and MyLC, components of the thick filament, as well as slow myosin binding protein-C (sMyBP-C), and the thin filament proteins troponin, tropomyosin, and nebulin [1,17]. Consequently, in the differential diagnosis of tremors, myogenic causes should be considered, particularly in cases without prominent myopathic features.

The mechanism of tremor generation in our patient remains unclear. Tremors can arise from both neurogenic and myogenic foci, and both are thought to result from oscillations within the servomechanism γ-loop associated with the stretch reflex. Central to this mechanism are the innervated intrafusal muscle fibers, whose activation in response to the magnitude and rate of changes in muscle length regulates the reflex arc. Given that MYH2 expression is muscle sarcomere-specific, we hypothesize that variants in *MYH2* could cause structural and/or contractile abnormalities at the sarcomere level, thereby inducing oscillations within the reflex arc and generating tremors.

Our case expands the clinical and mutational spectrum of *MYH2*-related myopathy and highlights that hand tremor, while rare, is a clinical feature that should not be overlooked in this disease. This insight underscores the importance of a comprehensive clinical evaluation and suggests that future research should focus on the under-recognized manifestations of *MYH2*-associated myopathy to improve early diagnosis and treatment strategies.

## References

[1] Schaefer J, Saak A, Bönnemann CG, Jackson S. Myogenic tremor – a novel tremor entity. Curr Opin Neurol. 2021 Oct; 34(5): 706–713. DOI: 10.1097/WCO.0000000000000976 34292195

[2] Wang L, Geist J, Grogan A, Hu LYR, Kontrogianni-Konstantopoulos A. Thick Filament Protein Network, Functions, and Disease Association. Compr Physiol. 2018 Apr; 8(2): 631–709. DOI: 10.1002/cphy.c170023 29687901 PMC6404781

[3] Ruppel KM, Spudich JA. Structure-function analysis of the motor domain of myosin. Annu Rev Cell Dev Biol. 1996; 12: 543–573. DOI: 10.1146/annurev.cellbio.12.1.543 8970737

[4] Lossos A, Oldfors A, Fellig Y, Meiner V, Argov Z, Tajsharghi H. *MYH2* mutation in recessive myopathy with external ophthalmoplegia linked to chromosome 17p13.1-p12. Brain. 2013 Jul; 136(7): e238–e238. DOI: 10.1093/BRAIN/AWS365 23388406

[5] Cassini TA, Malicdan MCV, Macnamara EF, Lehky T, Horkayne-Szakaly I, Huang Y, et al. *MYH2*-associated myopathy caused by a novel splice-site variant. Neuromuscul Disord. 2023 Mar; 33(3): 257–262. DOI: 10.1016/j.nmd.2022.12.014 36774715 PMC10023425

[6] Telese R, Pagliarani S, Lerario A, Ciscato P, Fagiolari G, Cassandrini D, et al. *MYH2* myopathy, a new case expands the clinical and pathological spectrum of the recessive form. Mol Genet Genomic Med. 2020; 8(9): 1–9. DOI: 10.1002/mgg3.1320 PMC7507101 32578970

[7] Martinsson T, Oldfors A, Darin N, Berg K, Tajsharghi H, Kyllerman M, et al. Autosomal dominant myopathy: missense mutation (Glu-706 → Lys) in the myosin heavy chain IIa gene. Proc Natl Acad Sci USA. 2000 Dec; 97(26): 14614–14619. DOI: 10.1073/pnas.250289597 11114175 PMC18967

[8] Jaganathan K, Kyriazopoulou Panagiotopoulou S, McRae JF, Darbandi SF, Knowles D, Li YI, et al. Predicting Splicing from Primary Sequence with Deep Learning. Cell. 2019; 176(3): 535–548. DOI: 10.1016/j.cell.2018.12.015 30661751

[9] Walker LC, Hoya M dela, Wiggins GAR, Lindy A, Vincent LM, Parsons MT, et al. Using the ACMG/AMP framework to capture evidence related to predicted and observed impact on splicing: Recommendations from the ClinGen SVI Splicing Subgroup. Am J Hum Genet. 2023 Jul; 110(7): 1046–1067. DOI: 10.1016/j.ajhg.2023.06.002 37352859 PMC10357475

[10] Schubach M, Maass T, Nazaretyan L, Röner S, Kircher M. CADD v1.7: using protein language models, regulatory CNNs and other nucleotide-level scores to improve genome-wide variant predictions. Nucleic Acids Res. 2024 Jan; 52(D1): D1143–D1154. DOI: 10.1093/nar/gkad989 38183205 PMC10767851

[11] Richards S, Aziz N, Bale S, Bick D, Das S, Gastier-Foster J, et al. Standards and guidelines for the interpretation of sequence variants: A joint consensus recommendation of the American College of Medical Genetics and Genomics and the Association for Molecular Pathology. Genetics in Medicine. 2015 May; 17(5): 405–424. DOI: 10.1038/gim.2015.30 25741868 PMC4544753

[12] Tajsharghi H, Darin N, Rekabdar E, Kyllerman M, Wahlström J, Martinsson T, et al. Mutations and sequence variation in the human myosin heavy chain IIa gene (*MYH2*). European Journal of Human Genetics. 2005; 13(5): 617–622. DOI: 10.1038/sj.ejhg.5201375 15741996

[13] Tajsharghi H, Hammans S, Lindberg C, Lossos A, Clarke NF, Mazanti I, et al. Recessive myosin myopathy with external ophthalmoplegia associated with *MYH2* mutations. Eur J Hum Genet. 2014; 22(6): 801–808. DOI: 10.1038/ejhg.2013.250 24193343 PMC4023224

[14] Maniyar AMH, Singh RK, Ojha PT, Chaudhary GS, Mahto AP, Shah AG. Myosin Myopathy Presenting as Chronic Progressive External Ophthalmoplegia. Ann Indian Acad Neurol. 2023; 26(6): 1024–1025. DOI: 10.4103/AIAN.AIAN_552_23 38229656 PMC10789400

[15] Tajsharghi H, Hilton-Jones D, Raheem O, Saukkonen AM, Oldfors A, Udd B. Human disease caused by loss of fast IIa myosin heavy chain due to recessive *MYH2* mutations. Brain. 2010; 133(5): 1451–1459. DOI: 10.1093/brain/awq083 20418530

[16] Darin N, Kyllerman M, Wahlström J, Martinsson T, Oldfors A. Autosomal dominant myopathy with congenital joint contractures, ophthalmoplegia, and rimmed vacuoles. Ann Neurol. 1998 Aug; 44(2): 242–248. DOI: 10.1002/ana.410440215 9708547

[17] Hauserman JG, Stavusis J, Joca HC, Robinett JC, Hanft L, Vandermeulen J, et al. Sarcomeric deficits underlie MYBPC1 associated myopathy with myogenic tremor. JCI Insight. 2021 Oct; 6(19). DOI: 10.1172/jci.insight.147612 PMC8525646 34437302

